# In vitro culture expansion impairs chondrogenic differentiation and the therapeutic effect of mesenchymal stem cells by regulating the unfolded protein response

**DOI:** 10.1186/s13036-018-0119-2

**Published:** 2018-11-20

**Authors:** Chong Shen, Tongmeng Jiang, Bo Zhu, Yiguan Le, Jianwei Liu, Zainen Qin, Haimin Chen, Gang Zhong, Li Zheng, Jinmin Zhao, Xingdong Zhang

**Affiliations:** 1 Guangxi Engineering Center in Biomedical Materials for Tissue and Organ Regeneration, Guangxi Collaborative Innovation Center for Biomedicine, Life Sciences Institute, Guangxi Medical University, Nanning, 530021 China; 2 Department of Orthopaedics Trauma and Hand Surgery, The First Affiliated Hospital of Guangxi Medical University, Nanning, China; 3 Guangxi Key Laboratory of Regenerative Medicine, International Joint Laboratory on Regeneration of Bone and Soft Tissue, The First Affiliated Hospital of Guangxi Medical University, Nanning, 530021 China; 4 National Engineering Research Center for Biomaterials, Sichuan University, Chengdu, 610064 China

**Keywords:** Endoplasmic reticulum stress, Unfolded protein response, Mesenchymal stem cells, Differentiation, Chondrogenesis

## Abstract

In vitro expansion of mesenchymal stem cells (MSCs) has been implicated in loss of multipotency, leading to impaired chondrogenic potential and an eventual therapeutic effect, as reported in our previous study. However, the precise regulatory mechanism is still unclear. Here, we demonstrate that endoplasmic reticulum (ER) stress and the unfolded protein response (UPR) were involved in transformation of MSCs induced by in vitro culture based on the comparative profiling of in vitro cultured bone marrow MSCs at passage 3 (P3 BMSCs) vs. fresh P0 BMSCs by microarray analysis. Indeed, RT-PCR and Western blot analysis showed significantly lower expression levels of three key UPR-related molecules, ATF4, ATF6 and XBP1, in P3 BMSCs than P0 BMSCs. Further, we found that UPR suppression by 4-phenylbutyrate (4-PBA) reduced the chondrogenic potential of P0 BMSCs and further cartilage regeneration. Conversely, UPR induction by tunicamycin (TM) enhanced the chondrogenic differentiation of P3 BMSCs and the therapeutic effect on cartilage repair. Thus, the decline in the chondrogenic potential of stem cells after in vitro culture and expansion may be due to changes in ER stress and the UPR pathway.

## Introduction

Mesenchymal stem cells (MSCs) are promising candidates for cell therapeutic approaches in regenerative medicine because of their multipotentiality. MSCs can be harvested from many tissues, including bone marrow and adipose tissue. To obtain sufficient cells for transplantation in clinical treatment, in vitro expansion is always a necessary step for MSCs derived from bone marrow [1]. However, long- [2–4] or short-term [5, 6] in vitro cultures lead to replicative senescence and impaired multipotency of stem cells. Using a hydrogel system in our previous study, we found decay in telomerase activity and changes in chromosomal anomaly in bone marrow-derived MSCs (BMSCs) at passage 3 (P3) compared to freshly isolated BMSCs (P0), thereby resulting in decreased chondrogenic potential of stem cells and a lower therapeutic effect in cartilage repair [7]. However, the precise mechanism underlying the impaired pluripotency of stem cells by in vitro expansion remains unknown.

Recently, it was found that endoplasmic reticulum (ER) stress caused by the accumulation of unfolded or misfolded proteins in the ER plays an important role during the process of stem cell differentiation under physiological or pathophysiological conditions [8, 9]. The increased protein unfolding and synthesis in the ER may be triggered by hypoxia, nutrient deprivation, perturbation of redox status, aberrant Ca^2+^ regulation, or failure of posttranslational modifications, for example [8, 10]. To alleviate the ER stress, the unfolded protein response (UPR) pathway is involved. During the process, *BIP*, a key ER chaperone binding to the transmembrane receptors of the three UPR sensors *PERK*, *IRE1*, and *ATF6*, is released, leading to activation of the UPR pathway [11–13]. Mediation of the UPR pathway has been reported to be associated with cell differentiation, including adipogenesis, osteogenesis and chondrogenesis [14–18]. Repression of UPR prevented the differentiation of embryonic stem cells (ESCs) into definitive endodermal cells, while induction of UPR enhances the endodermal differentiation of ESCs [19]. The major UPR sensors also mediate the differentiation of stem cells. Overexpression of *ATF6* activates chondrogenesis, whereas knockdown of *ATF6* abolishes chondrogenesis differentiation and endochondral bone growth [16]. The *IRE1* downstream mediator *XBP1* prominently functions in chondrogenic differentiation of MSCs, whereas reductions in *XBP1* expression decrease chondrogenic differentiation [17]. Downregulation of *ATF4*, a downstream mediator of *PERK*, mitigates the expression of cartilage markers [18]. These findings suggested that ER stress and the UPR pathway play crucial roles in stem cell differentiation.

Preliminary analysis based on the comparative profiling of P3 BMSCs vs. P0 BMSCs in our previous study [7] showed that ER stress and UPR signaling were significantly different, demonstrating their association with in vitro culture. In this study, we investigated the effect of ER stress and changes in the UPR on the chondrogenic potential of BMSCs in vitro and BMSCs-based cartilage regeneration in vivo using the ER stress inhibitor (4-phenylbutyrate (4-PBA)) in P0 BMSCs and the ER stress inducer (tunicamycin (TM)) in P3 BMSCs.

## Materials and methods

### BMSC isolation and in vitro expansion

Sixty male New Zealand white rabbits (Experimental Animal Centre of the Guangxi Medical University, Nanning, China) weighing 2.5–3 kg and aged 2 months were used**.** The experiments were approved by the Animal Care and Experiment Committee of Guangxi Medical University (protocol number: 2014-12-3). After the rabbits were anaesthetized with pentobarbital, a 16 G needle was used to puncture the bilateral femurs and tibias, and approximately 20 ml of bone marrow was aspirated. BMSC were harvested using a bone marrow mononuclear cell isolation kit (TBD2013CRA, Tian Jin Hao Yang Biological Manufacture Co., Ltd., Tianjin, China). BMSCs were separated into four groups as follows: (1) the P0 group: fresh BMSCs were isolated from the bone marrow of untreated rabbits. (2) The P0 + 4-PBA group: fresh BMSCs were isolated from the bone marrow of rabbits which had been treated with 0.25 g/kg of 4-PBA (Sigma, St. Louis, MO, USA) by oral gavage once a day for 20 days. The dosage of 4-PBA was based on a published report [20]. (3) The P3 group: P0 BMSCs were cultured and expanded up to passage 3 in vitro*.* (4) P3 + TM group: P3 BMSCs were cultured with 0.25 μg/ml of TM (Sigma, St. Louis, MO, USA) for 48 h, as previously reported [19, 21].

### Cell seeding

Neutralized type I collagen solution was prepared as described in previous studies [22–24]. BMSCs were seeded in a collagen solution at a density of 1 × 10^7^ cells/ml. The mixture of BMSCs and collagen solution was gelated through physical crosslinking at 37 °C for 10 min. Then, they were cultured in chondrogenic medium supplemented with 10 ng/ml of TGF-β1 (PeproTech, Rocky Hill, PA, USA), 50 μg/ml ascorbic acid (Sigma), 100 nM dexamethasone (Sigma) and 1% insulin-transferrin-selenium solution (Gibco) for 7, 14 or 21 days. The medium was changed every 3 days.

### Microarray analysis and data processing

Total RNA was extracted from the original P0 and P3 samples using TRIzol reagent (Invitrogen). Microarray analysis was performed on an Agilent Array platform by Shanghai KangChen Biotech in three replicates. The purity and concentration of RNA were measured using the NanoDrop ND-1000 spectrophotometer (NanoDrop Technologies, Hudson, NH, USA). RNA integrity was determined via denaturing gel electrophoresis. The following procedures were performed according to the Agilent Whole Genome Oligo Microarray (one-color) protocol.

Array data preprocessing, normalization and quality control were conducted using GeneSpring software V12.1 (Agilent Technologies). A *P* < 0.05 and an absolute log base 2 fold change greater than 1 were considered as the criteria for differentially expressed gene selection. The differentially expressed genes (DEGs) were identified using the Significance for microarrays (SAM) graph through the MultiExperiment Viewer (MeV 4.9). KEGG pathway analysis was applied to determine key signaling pathways and relationships between these differentially expressed genes. The resulting data were Log base 2 transformed and subjected to further analysis by hierarchical clustering with average linkage by CLUSTER 3.0 (Stanford University School of Medicine, Stanford, CA, USA). Finally, a heat map was generated and visualized using Java Treeview software (Stanford University School of Medicine, Stanford, CA, USA).

### Quantitative real-time PCR

Total RNA was isolated using an RNA isolation kit (Tiangen Biotechnology, Beijing, China) and reverse transcribed into cDNA using a reverse transcription kit (Takara, Japan) following the manufacturer’s instructions. Real-time PCR was performed using Fast Start Universal SYBR Green Master Mix (Roche, Germany) and a Light Cycle 96 system for 10 min at 95 °C, 15 s at 95 °C, and 1 min at 60 °C. The expression levels of target genes were normalized to GAPDH expression. The results were analyzed using the 2^–ΔΔ^CT method. The primer sequences are summarized in Table 1.

**Table 1:** Primer sequences used in qRT-PCR experiments

mRNA	Forward primer	Reverse primer
*XBP1*	CTGGAACAGCAAGTCGTGGA	GCTGCAGATGCACGTAGTCT
*ATF4*	AGTGGACCTCAAGGAGTTCG	CAAGCTGAACGACTCATCCG
*ATF6*	GCAAACCAGAGGAGGCATCT	CCTGAGCGACTCTGTTGTGT
*ACAN*	TTGCCTTTGTGGACACCAGT	GAGCCAAGGACGTAAACCCA
*SOX9*	GACGCACATCTCGCCCAAC	TCTCGCTTCAGGTCAGCCTT
*COL2A1*	ACTGGTGGAGCAGCAAGAGC	GACGTTGGCAGTGTTGGGAG
*COL1A1*	GCCACCTCAAGAGAAGGCTG	CTCGGGGCTCTTGATGTTCT
*GAPDH*	GTCATCATCTCAGCCCCCTC	GGATGCGTTGCTGACAATCT

### Western blot analyses

Total proteins were extracted using RIPA Lysis Buffer (Beyotime, Beijing, China) and phenylmethanesulfonyl fluoride (PMSF) (Beyotime, China). The proteins were separated in sodium dodecyl sulfate (SDS-PAGE) and electroblotted onto PVDF membranes. The membranes were incubated overnight at 4 °C with appropriate primary antibodies against β-Actin (Boster, Wuhan, China), COL2A1 (Boster), XBP1 (Sigma, USA), ATF6 (Sigma) or ATF4 (Bioss, Beijing, China) after incubation in 1% BSA blocking buffer (Boster) for 1 h at room temperature. Immunoreactive signals were detected by secondary antibodies (Invitrogen, Carlsbad, CA, USA) using the Odyssey Infrared Imaging System (LI-COR).

### Cell proliferation analysis

The Cell-gel pellets were transferred to a 24-well plate after 7, 14 or 21 days of culture. Proliferation of BMSCs was detected by the MTT assay. First, 500 μL of MTT reagent was added to each well and incubated for 4 h. The supernatant was discarded and 500 μL of dimethylsulfoxide was added and incubated for an additional 2 h. The cells were then transferred to 2 mL centrifuge tubes and destroyed using forceps and sonication. The supernatant solution was collected and the absorbance was measured by a Microplate Reader (Thermo Fisher Scientific, Waltham, MA, USA) at 570 nm.

### Glycosaminoglycan detection

Cell-gel pellets were washed with PBS and digested with 60 μg/ml proteinase K (Sigma) for 10 h at 56 °C after 7, 14 or 21 days of culture. The digested aliquots were assayed separately to assess their GAG and DNA content. The GAG content was measured using the dimethylmethylene blue dye binding assay and the absorbance was measured at 525 nm using a microplate reader (Thermo, Karlsruhe, Germany). The cellularity was measured based on the DNA content using Hoechst 33258 (Sigma) and the fluorescence intensity was measured at 460 nm using a microplate fluorescence reader (FLX800, Bio-tec, Burlington, Vermont, USA). The DNA and glycosaminoglycan (GAG) contents of the pellets were quantified and GAG production was expressed as the GAG/DNA ratio. Safranin-O staining was conducted to evaluate the GAG production after 21 days of chondrogenic differentiation as well.

### Immunofluorescence

BMSCs were seeded on glass coverslips in 24-well plates and incubated overnight. The next day, they were fixed with 4% paraformaldehyde for 30 min and then permeabilized with 0.1% Triton X-100 for 10 min at room temperature. To block endogenous peroxidase and prevent nonspecific binding, fixed cells were successively treated with 3% H_2_O_2_ for 10 min and 5% normal goat serum for 10 min at room temperature. Cell samples were incubated at 4 °C overnight with primary antibody as follows: XBP1 (1:200, Sigma), ATF6 (1:200, Sigma) and ATF4 (1:400, Bioss, Bei Jin, China). The samples were then incubated with the appropriate secondary antibodies (Alexa Fluor) for 45 min at 37 °C and counterstained with 4′, 6-diamidino-2-phenylindole (DAPI) for 5 min at room temperature. Immunofluorescent staining for COL2A1 (1:100, Boster, China) was also performed for BMSCs after 21 days of chondrogenic induction. Images were taken with a fluorescence microscope (Olympus) after slides were mounted with fluorescent mounting media (Dako).

### Animal model and surgical procedure

Both knees of the rabbits were subjected to the operation. After general anesthesia, a lateral parapatellar approach was used to expose the knee and a chondral-only defect of 4.0 mm in diameter was created in the medial area of each patellar groove. Then, collagen hydrogel loaded with BMSCs was injected into the defective area. The defects were filled as follows: (1) collagen hydrogel with P0 BMSCs (P0 group, *n* = 30 knees); (2) collagen hydrogel with P0 BMSCs + 4-PBA, which from rabbits were fed after 20 days of administration with 4-PBA (P0 + 4-PBA group, *n* = 30 knees); (3) collagen hydrogel with P3 BMSCs (P3 group, n = 30 knees); and (4) collagen hydrogel with P3 BMSCs +TM, which were treated with TM for 48 h (P3 + TM group, n = 30 knees). Rabbits were euthanized by injection with an overdose of pentobarbital sodium at 4, 12, and 26 weeks after surgery and the repaired cartilage was used for further analysis.

### Gross analysis and grading

The gross morphological evaluation of the defect repairs were carried out according to the International Cartilage Repair Society (ICRS) scoring system, which encompasses four major categories; the total score was 24 [25].

### Mechanical evaluation

Mechanical evaluation was performed as described in previous studies [26]. In brief, the compressive mechanical properties of the repaired tissues were tested by a compression strength tester (model HY-0230; Shanghai Hengyi Instruments Co., Ltd., Shanghai, China). A 2 mm diameter cylindrical indenter fitted with a 10 N maximum loading cell was selected. A step displacement (20% strain) was determined to apply and monitor the compressive force over time until unconfined equilibrium was reached. The crosshead speed was approximately 0.06 mm/min. The ratio of equilibrium force to cross-sectional area was divided by the applied strain to calculate the equilibrium modulus (in MPa). Samples were evaluated at 12 and 26 weeks postoperation, *n* = 6 knees/ (group, time point).

### Histological and immunohistochemical examination

The osteochondral blocks containing the defects were fixed in 10% neutral buffered formalin and subsequently decalcified with a 14% ethylenediaminetetraacetic acid (EDTA) solution for at least two weeks. The blocks were embedded in paraffin and cut into 5 μm thick slices using a sharp blade. Safranin O-fast green staining for histomorphological analysis and immunohistochemical staining for COL2A1 (1:100, Boster) were performed to evaluate cartilage regeneration according to standard protocols. Microphotographs were taken using a light microscope (Olympus BX53, Tokyo, Japan). The repaired tissues were graded blindly by three different researchers using the ICRS Visual Histological Assessment Scale [27].

### Statistical analysis

Statistical comparisons were made using Student’s T- test between two samples, and one-way analysis of variance (ANOVA) was used to compare the means among different groups. Tukey’s test was used in the post hoc multiple comparisons. All data are presented as the mean ± SD, and a *P* value of less than 0.05 was considered significant.

## Results

### Comparative analysis of gene expression profiles of P0 and P3 BMSCs

**Fig. 1:**
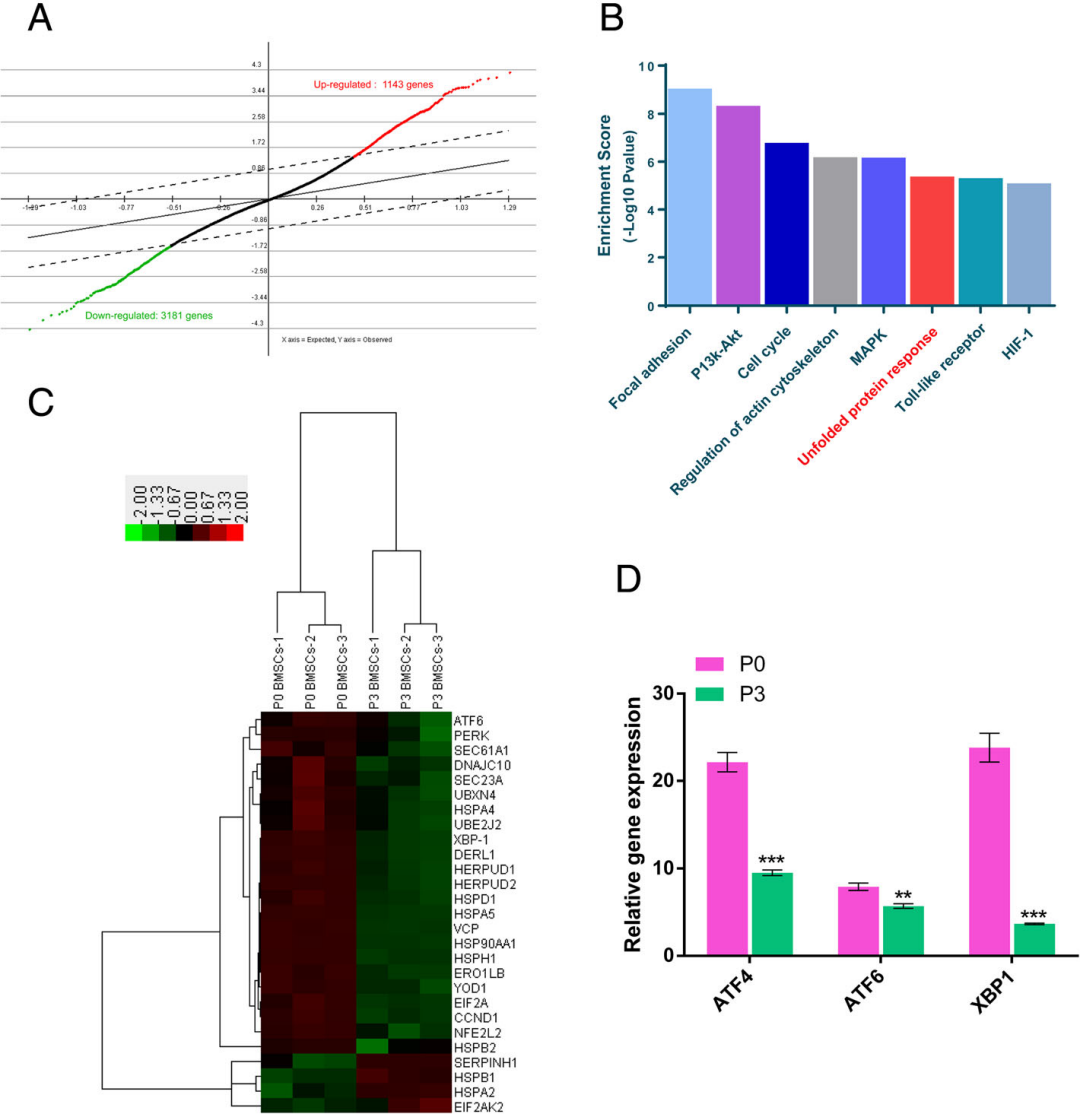
Microarray and quantitative RT-PCR analysis of P0 and P3 BMSCs. **a** Differential gene expression in P3 BMSCs compared with P0 BMSCs. **b** Pathway enrichment analyses of differentially expressed genes between P3 and P0 BMSCs. **c** Hierarchical cluster analysis of UPR-related genes showed differential expression between P3 and P0 BMSCs. The genes connected by the lines indicate the clustered genes based on Hierarchical cluster analysis. **d** Expression of the three critical genes (*ATF4*, *ATF6*, and *XBP1*) of UPR was measured by quantitative RT-PCR analysis in P0 and P3 BMSCs. These data were normalized to *GAPDH*. The values were means ± S.D.; * indicate *P* < 0.05, ** indicate *P* < 0.01, *** indicate *P* < 0.001

To unravel the underlying mechanism of genetic alteration caused by in vitro expansion, we compared global gene expression signatures between P0 BMSCs and P3 BMSCs using a microarray analysis. There were 1143 upregulated and 3181 downregulated transcripts in the P3 BMSCs compared with the P0 BMSCs (Fig. 1 a). By pathway enrichment analyses, we found differentially expressed genes (DEGs) involved in several pathways, including focal adhesion, PI3K-Akt, cell cycle, regulation of actin cytoskeleton, MAPK, UPR, toll-like receptor, and HIF-1 (Fig. 1 b). Based on hierarchical clustering analysis, we found that most genes involved in UPR were downregulated in P3 BMSCs (Fig. 1 c).

To validate gene expression levels in microarray analysis, several established UPR-associated markers were assessed by quantitative RT-PCR analysis. As shown in Fig. 1 d, three UPR sensors - *ATF4*, *XBP1*, and *ATF6* - were significantly decreased in P3 BMSCs compared to P0 BMSCs (*P* < 0.05), which verified the microarray analysis. It is noteworthy that the expression of *ATF6* also decreased significantly in the P3 group compared with P0 (*P* < 0.05), although it is not significantly downregulated as shown in the microarray analysis (*P* > 0.05).

### ER stress inhibitor reduced UPR in P0 BMSCs while ER stress inducer promoted UPR in P3 BMSCs

**Fig. 2:**
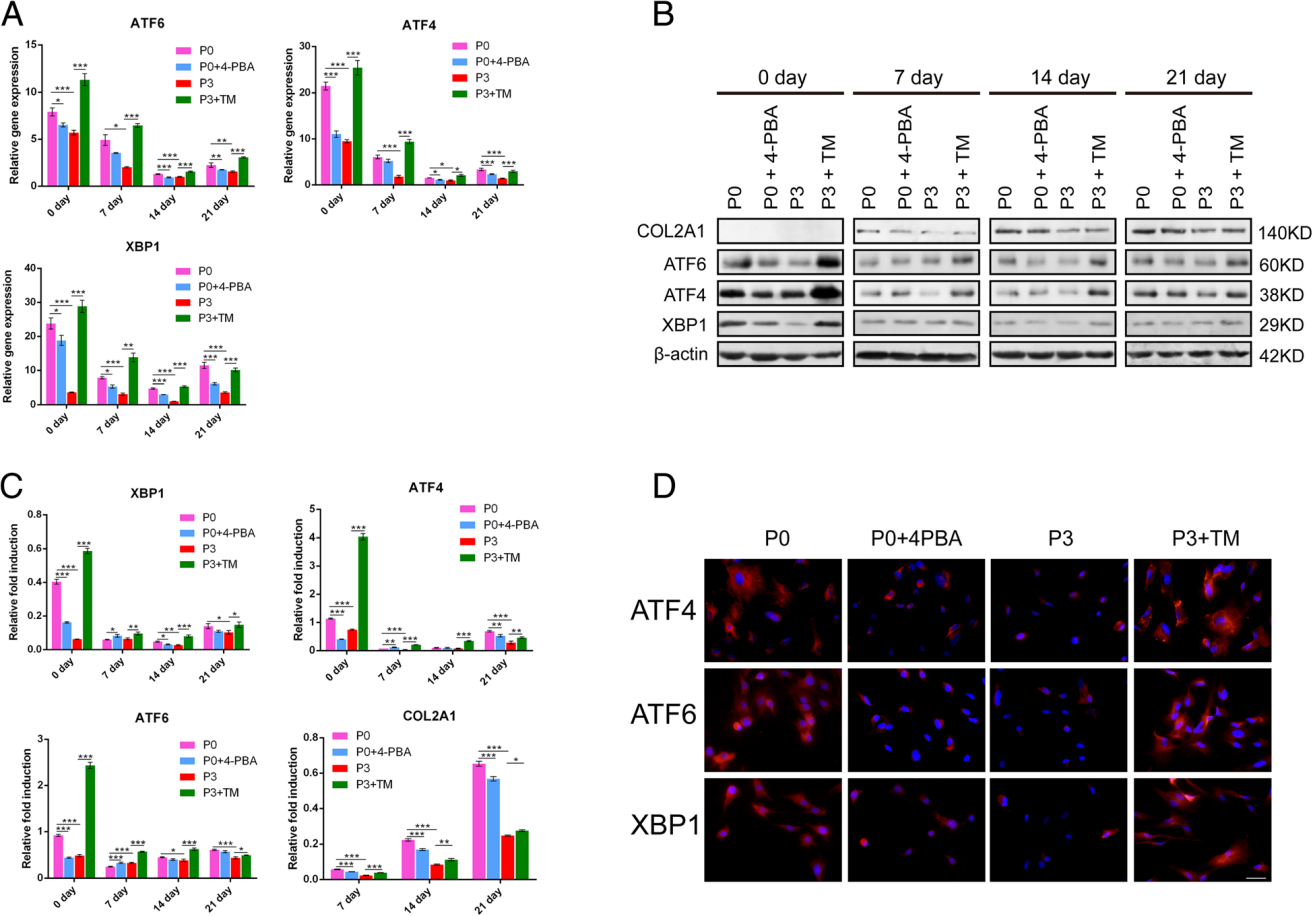
UPR-related gene/protein expression in four groups. **a** Quantitative RT-PCR for *ATF6*, *ATF4*, and *XBP1* of the four groups in the cultured constructs on 0, 7, 14, and 21 days. These data were normalized to *GAPDH*. **b** Western blot results for ATF4, ATF6, XBP1, and COL2A1 at different culture times. COL2A1 was not detectable at 0 day; β-actin was used as a loading control. **c** Normalized expression of ATF4, ATF6, XBP1, and COL2A1 in response to Western blot analysis. **d** Immunofluorescence staining of ATF4, ATF6, and XBP1 at 0 days. Scale bar: 50 μm. The values were means ± S.D.; * indicate *P* < 0.05, ** indicate *P* < 0.01, *** indicate *P* < 0.001

To investigate the effect of the UPR pathway on BMSCs differentiation, we introduced P0 BMSCs from rabbits treated with the ER stress inhibitor 4-PBA [20, 28] for 20 days and P3 BMSCs treated with the ER stress inducer TM [29] besides the original P0 and P3 BMSCs groups. As shown in Fig. 2 a, mRNA expression of *ATF4*, *ATF6* and *XBP1* in the UPR pathway was particularly high at the initial time and almost gradually decreased with time in all groups. The gene expression of *ATF4*, *ATF6* and *XBP1* was markedly lower in P3 BMSCs than in P0 BMSCs at each time point (*P* < 0.05). After treatment with 4-PBA in P0 BMSCs, the expression of the three genes was significantly downregulated (*P* < 0.05). In contrast, the expression of *ATF4*, *ATF6* and *XBP1* was significantly increased in P3 BMSCs after treatment with TM (*P* < 0.05, Fig. 2 a). Western blotting analysis confirmed that protein expression of ATF6, ATF4 and XBP1 was correspondingly reduced in P0 + 4-PBA compared with P0 and increased in P3 + TM compared with P3 (Fig. 2 b, c). Immunofluorescence staining showed that there were more positive stain areas for ATF4, ATF6, and XBP1 in the P0 and P3 + TM groups than the P0 + 4-PBA and P3 groups (Fig. 2 d), which is in accordance with the results of quantitative RT-PCR and Western blotting. These findings suggested that the chemical chaperone ER stress inhibitor 4-PBA successfully suppressed UPR in P0 BMSCs, and the ER stress inducer TM effectively induced ER stress and UPR in P3 BMSCs.

### ER stress and UPR inhibition reduced chondrogenic differentiation in P0 BMSCs while ER stress and UPR induction promoted chondrogenic differentiation in P3 BMSCs

**Fig. 3:**
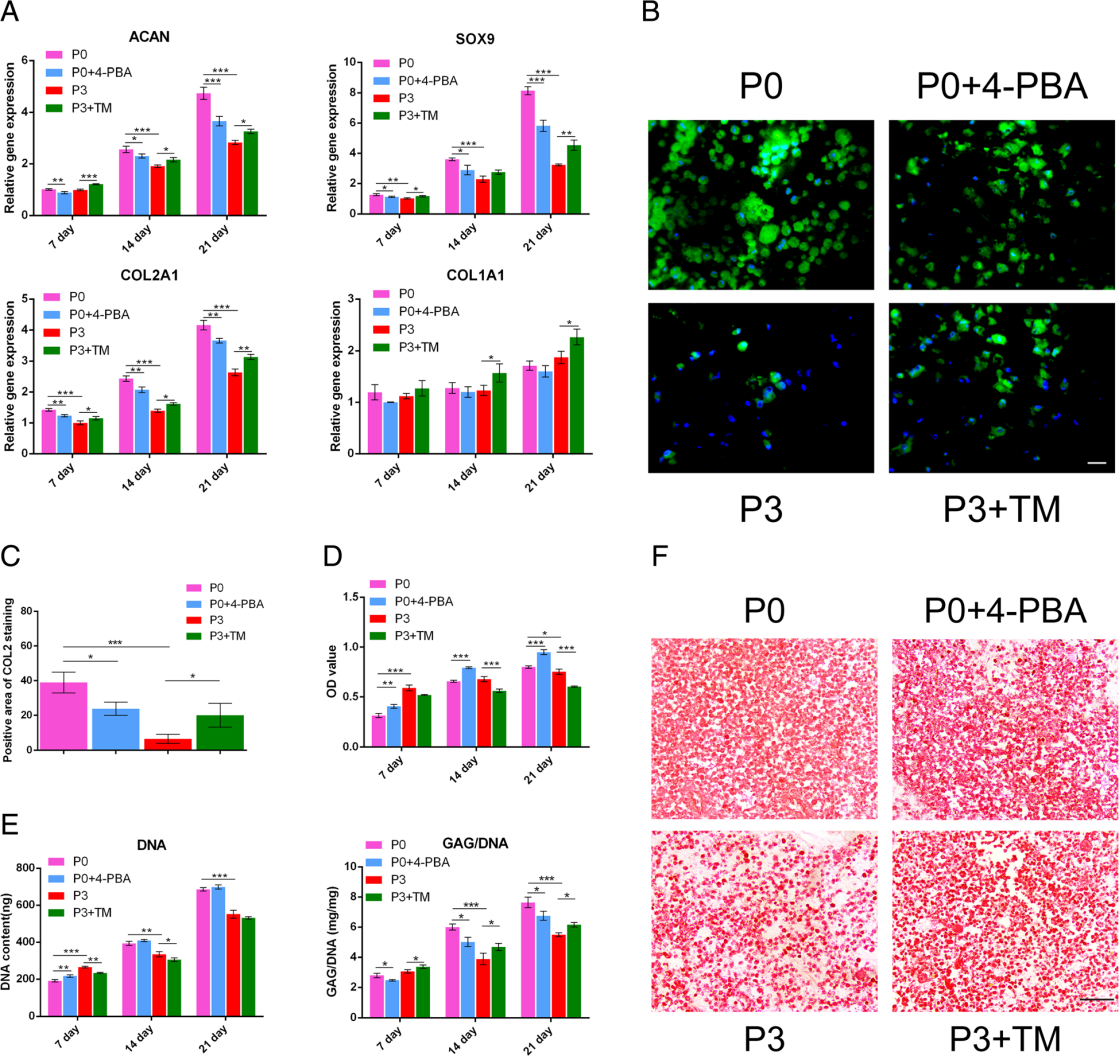
Chondrogenic-related gene/protein expression in four groups. **a** Quantitative RT-PCR for *ACAN*, *SOX9*, *COL2A1*, and *COL1A1* of the four groups in the cultured constructs on 7, 14, and 21 days. These data were normalized to *GAPDH*. **b** Immunofluorescence staining of COL2A1 after 21 days of chondrogenic induction. Scale bar: 25 μm. **c** The semi-quantitative analysis of positive staining of COL2A1 in response to Fig. 3 b. **d** MTT assay was used to analyze cell proliferation of the four groups: P0 BMSCs, P0 + 4-PBA, P3 BMSCs and P3 + TM. **e** The DNA and GAG contents were stained for the Hoechst 33258 and dimethylmethylene blue dye binding assays, respectively, after 7, 14 and 21 days of chondrogenic induction, and the absorbances were measured to quantify the contents of DNA and GAG. The left panel shows the DNA content and the right panel shows the ratio between GAG and DNA content. **f** GAG production was examined using Safranin-O staining in the four samples: P0 BMSCs, P0 with 4-PBA treatment, P3 BMSCs and P3 with TM treatment at 21 days. Scale bar: 100 μm. The values are means ± S.D.; * indicate *P* < 0.05, ** indicate *P* < 0.01, *** indicate *P* < 0.001

To further uncover the effect of ER stress and UPR variation on chondrogenic differentiation in BMSCs, we assessed the mRNA expression level of cartilage-specific markers, including *ACAN*, *SOX9* and *COL2A1,* by quantitative RT-PCR analysis (Fig. 3 a). We found that the expression of cartilage-specific genes was increased in a time-dependent manner in all the groups. In comparison, expression levels of the cartilage specific markers were significantly lower in P3 BMSCs than in P0 BMSCs (*P* < 0.05). Moreover, 4-PBA administration significantly downregulated the expression of the cartilage markers in P0 BMSCs (*P* < 0.05), while TM treatment promoted the gene expression (*P* < 0.05). Of note, expression of *COL1A1*, a fibrocartilage marker, is comparable between the P0 and P0 + 4-PBA groups at all indicated times. It is slightly upregulated in P3 + TM at 14 and 21 days compared with P3 (Fig. 3 a). As shown in the Western blotting analysis, protein expression of COL2A1, which is the hyaline cartilage marker, was decreased significantly in the P0 + 4-PBA group compared with the P0 group at different time points (*P* < 0.05) (Fig. 2 b, c). Meanwhile, its expression was significantly elevated in the P3 + TM group compared with the P3 group (*P* < 0.05) (Fig. 2 b, c), in agreement with the quantitative RT-PCR findings. This was confirmed by immunofluorescence staining of COL2A1 and semi-quantitative analysis measured after 21 days. As shown in Fig. 3 b and c, P0 BMSCs showed intense positive staining but P3 BMSCs exhibited almost negative staining. 4-PBA administration resulted in slightly positive staining, which was much weaker than P0 BMSCs. TM led to enhanced positive staining in P3 BMSCs. Taken together, these data suggested that ER stress and UPR suppression impaired the chondrogenic differentiation ability of P0 BMSCs, while ER stress and UPR activation promoted chondrogenic differentiation of P3 BMSCs.

To evaluate the effect of UPR variation on the proliferation of BMSCs, we performed the MTT assay and measured DNA content of BMSCs. As shown as Fig. 3 d and e, the OD value and DNA content increased gradually from 7 to 21 days in all groups, indicating that cell proliferation of BMSCs was time-dependent. Both 4-PBA and TM have minimal cytotoxicity to BMSCs, as evidenced by no obvious change in DNA content after treatment. GAG production, which marks the secretion of cartilage matrix, increased over time in all the groups. GAG content was lower in P0 + 4-PBA compared with P0 (*P* < 0.05) but was higher in P3 + TM vs. P3 BMSCs (*P* < 0.05). This was also confirmed by safranin-O staining, with more positive staining in P0 BMSCs than P0 + 4-PBA and in P3 + TM than P3, indicating that 4-PBA was associated with a reduction in UPR, inhibiting the synthesis of chondrogenic glycosaminoglycan in P0 BMSCs, and TM-mediated UPR induction accelerated the chondrocytic matrix in P3 BMSCs (Fig. 3f ). Taken together, these results suggested that ER stress and changes in the UPR influenced chondrogenic differentiation in BMSCs cultured in vitro.

### ER stress and UPR reduction impaired the therapeutic effect of BMSCs on cartilage defects

**Fig. 4:**
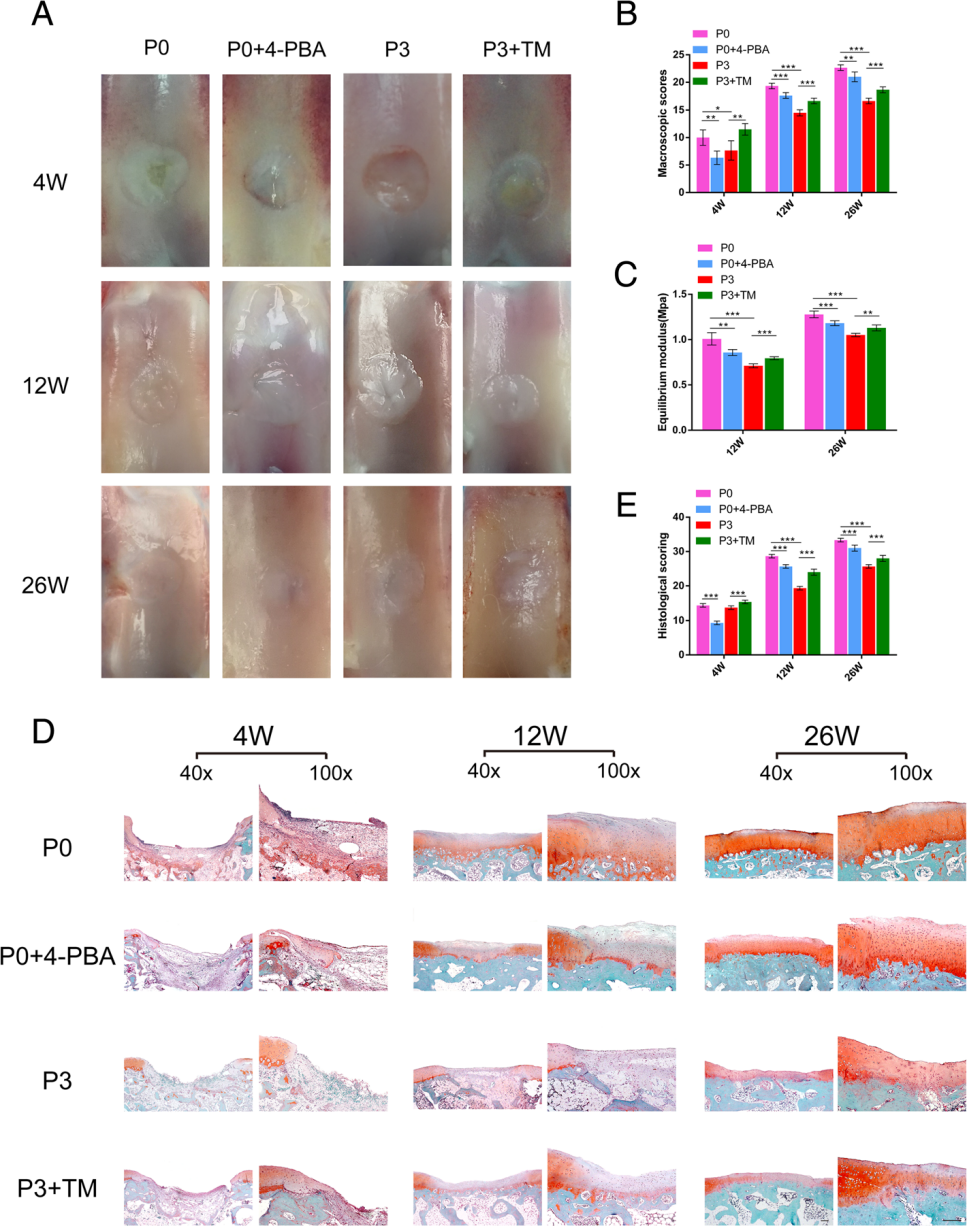
Therapeutic effect of BMSCs with different ER stress levels on cartilage defects. **a** Macroscopic appearance in cartilage defect healing in the four groups at 4, 12, and 26 weeks after surgery. **b** ICRS macroscopic scores for all samples from the four groups at different times after surgery (Fig. 4 a). **c** Biomechanical testing was performed at 12 weeks and 26 weeks after surgery. **d** Safranin O-fast green staining of repaired cartilage in vivo at each time point, respectively. Scale bar: 200 μm. **e** Histological scores for the samples from the four groups according to the ICRS Scale (Fig. 4 d). The values were means ± S.D.; * indicate *P* < 0.05, ** indicate *P* < 0.01, *** indicate *P* < 0.001

To further examine the effect of ER stress and UPR change on BMSCs-based therapy for cartilage defects, we created a cartilage defect model by creating a 4-mm diameter chondral defect in the patellar groove of rabbits and injecting collagen hydrogel with treated or untreated BMSCs. We monitored the repair efficacy of the cartilage defect by macroscopic assessment. As expected, the defects were partially filled with white tissue, with rough surfaces and obvious boundaries, 4 weeks after the operation in all groups. Twelve weeks after implantation of BMSCs, the regenerated tissue had filled the defects completely with visible boundaries in all groups. After 26 weeks, the defects were filled with white tissue and the interface was hardly discernable (Fig. 4a). In the P0 group, the neo-tissue showed the best integration with the surrounding tissue in all the groups, which scored the highest (Fig. 4 b). However, ER stress and UPR reduction by 4-PBA interference led to an impaired therapeutic effect with a 92% lower score. In the P3 group, the defects remained filled with white opaque tissue and had rough surfaces and obvious boundaries that scored 16.67; TM increased the score to 18.67 (Fig. 4b).

Biomechanical evaluation revealed that the stiffness of the regenerated tissues in the four groups increased dramatically from 12 to 26 weeks. Stiffer neo-cartilages were present in the P0 groups than the P3 groups. It is interesting that 4-PBA treatment lead to softer neo-issue in P0 BMSCs, while TM slightly promoted the mechanical strength of newly formed cartilage after 12 and 26 weeks (Fig. 4 c).

**Fig. 5:**
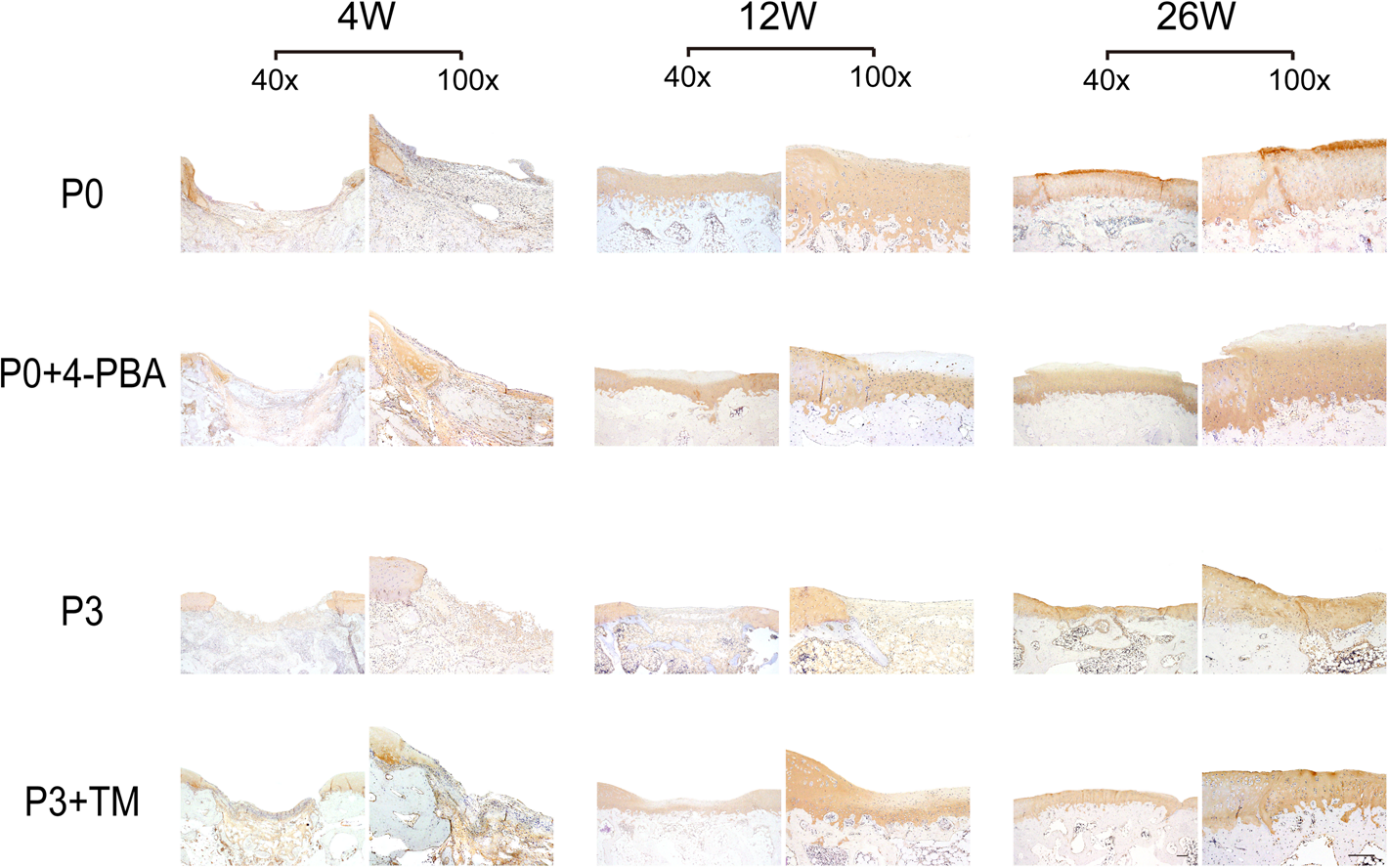
Immunohistochemical staining of COL2A1 in vivo at indicated times postoperation. Scale bar: 200 μm

To further evaluate the effect of ER stress and UPR change on cartilage regeneration, we performed histological and immunohistochemical staining. After 4 weeks of therapy, the defects showed significant depression and were mostly filled with fibrous tissue and incompletely differentiated BMSCs, with negative staining by safranin-O in all groups. After 12 weeks, the reconstructed tissue gradually progressed from fibrous tissue to fibrous cartilage-like tissue. After 26 weeks, the regenerated tissue was almost hyaline cartilage-like, with round chondrocytes embedded in the lacunae and accumulative GAG production, as indicated by intense positive staining (Fig. 4d). At each time point, the P0 BMSCs therapy group showed a better therapeutic effect than the others, as evidenced by more intense positive staining and more round cells. In contrast, the P3 groups had lower scores than the other groups at 12 weeks and 26 weeks of treatment. 4-PBA treatment decreased GAG production and the amount of round cells in P0 BMSCs therapy with a 35% (4 W), 10% (12 W), and 7% (26 W) decrease in scoring. TM treatment augmented hyaline cartilage-like tissue regeneration and GAG secretion in P3 BMSCs therapy with an 11% (4 W), 19% (12 W) and 8% (26 W) increase, indicating that the initial ER stress interference has a potent impact on the long-term regeneration of neo-cartilage (Fig. 4e). Immunohistochemical staining of type II collagen, which is hyaline cartilage marker, also confirmed the histological findings. The regenerated tissue progressed from negative staining to positive staining over time in all the groups. At each time point, secretion of type II collagen decreases in the order of P0, P0 + 4-PBA, P3 + TM and P3, revealing that the ER stress inhibitor 4-PBA delays cartilage restoration while the ER stress enhancer TM accelerates healing (Fig. 5).

## Discussion

In our study, we demonstrated that in vitro expansion impaired the differentiation capability of stem cells, possibly by suppression of ER stress and UPR pathways.

After a short-period monolayer culture in vitro, P3 BMSCs had a significantly lower level of UPR sensors (ATF6, ATF4, and XBP1) compared to freshly isolated P0 BMSCs. After a 21-day 3D culture, the level of UPR sensors dropped dramatically in all the groups. These findings suggested an association of the ER stress anomaly with impaired stem cells by in vitro culture. As we have known, ER homeostasis is critical for stem cell function in vivo [9]. Extensive increase or decrease in ER stress disrupts the balance of bone homeostasis and physiology [30]. BMSCs adapt themselves in a native hypoxic microenvironment in vivo [31–33], which always serves as an important stimulus of ER stress [34]. BMSCs had impaired chondrogenic potential after isolation and culture under normoxic conditions in vitro [35, 36]*,* in accordance with the change in ER stress. ATF4, an indicator of oxygen metabolism in cells [37, 38], was downregulated in P3 BMSCs compared with P0 BMSCs, suggesting the change in the oxidative microenvironment and ER stress [39] by in vitro expansion.

Interestingly, we found that the attenuation of ER stress and UPR resulted in decreased chondrogenic capability of BMSCs, as evidenced by 4-PBA interference in the P0 + 4-PBA group. Our finding is consistent with those of Xu [19], who reported an effect of UPR induction on endodermal differentiation in mouse embryonic stem cells (ESCs) when using the ER stress inhibitor tauroursodeoxycholic acid (TUDCA) to prevent the differentiation of ESCs into definitive endodermal cells. Meanwhile, the ER stress inducer tunicamycin or thapsigargin enhanced endodermal differentiation of ESCs by regulating the Smad2 and β-catenin pathways. Similarly, it was found that adipogenesis was closely related to ER stress and UPR. Knockdown of *XBP1* or blocking the UPR pathway by 4-PBA or TUDCA inhibited adipogenesis in vitro and in vivo [14, 40]. Moreover, knockdown of UPR sensors (*ATF6*, *ATF4*, and *XBP1*) decreased the expression of cartilage markers during chondrogenic differentiation of stem cells [16–18], which mirrors our findings.

On the other hand, induction of ER stress and UPR by TM promoted chondrogenic differentiation of BMSCs in the P3+ TM group by interference. Activation of ER stress and UPR is a very early critical step in cell differentiation [41]. In neuronal differentiation, the induction of ER stress and UPR in BMSCs or ESCs by tunicamycin or thapsigargin resulted in upregulation of the neuronal marker neurofilament-L and neurofilament-M [21]. Similarly, UPR improves the differentiation of neural precursor cells (NPCs) into astrocytes through the temporally controlled transcription factor Gcm1. Treatment of neural precursor cells with low doses of several ER stress inducers, such as tunicamycin and dithiothreitol, promotes NPCs differentiation toward astrocytes [42]. Induction of ER stress in oesophageal epithelial cells with thapsigargin also induces differentiation and the prevention of oesophageal cancer development [43]. Moreover, upregulation of UPR sensors (*ATF6*, *ATF4*, and *XBP1*) enhances the chondrogenic differentiation during stem cell differentiation [16–18].

However, there are some limitations to this study. The impacts of hypoxia on ER stress and UPR were not explored, and further influences on BMSCs should be studied in the future. Moreover, the interaction of hypoxia and in vitro culture was not fully investigated.

**Fig. 6:**
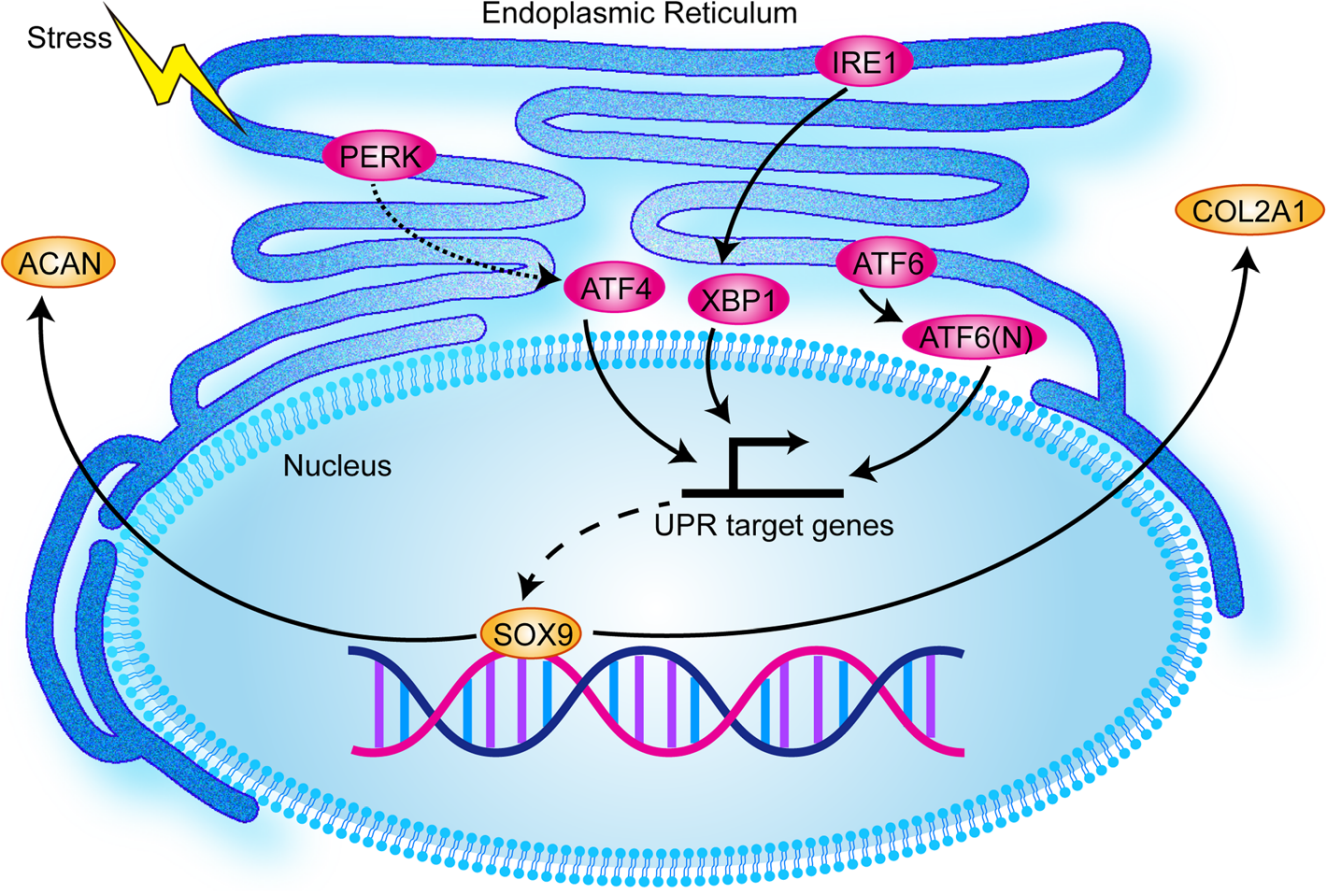
Schematic of chondrogenic differentiation of MSC through UPR induction

In conclusion, we showed that the decline in chondrogenic potential of stem cells after in vitro culture and expansion may be mediated by ER stress and the UPR pathway (Fig. 6). Activation of ER stress and UPR promoted chondrogenesis in expanded BMSCs, favorable for therapy of cartilage defects. In addition, their inhibition weakened the chondrogenic differentiation of freshly isolated BMSCs and was unfavorable to cartilage regeneration.
